# The Essential Role of Vitamin D in Pediatric Health: Implications for Oropharyngeal Infections and Neutropenia-Associated Vulnerability

**DOI:** 10.3390/children12091116

**Published:** 2025-08-25

**Authors:** Felicia Manole, Evelin Claudia Ghitea, Marc Cristian Ghitea, Timea Claudia Ghitea, Alexia Manole

**Affiliations:** 1 Clinical Department, Faculty of Medicine and Pharmacy, University of Oradea, 410068 Oradea, Romania; fmanole@uoradea.ro; 2 Faculty of Medicine and Pharmacy, University of Oradea, 410068 Oradea, Romania; ghitea.evelinclaudia@student.uoradea.ro (E.C.G.); ghitea.marccristian@student.uoradea.ro (M.C.G.); alexia.manole@csud.uoradea.ro (A.M.); 3 Pharmacy Department, Faculty of Medicine and Pharmacy, University of Oradea, 410068 Oradea, Romania

**Keywords:** vitamin D, health, disease, inflammation, vitamin D deficiency

## Abstract

**Highlights:**

**What are the main findings?**

Vitamin D deficiency was significantly associated with recurrent oropharyngeal infections (nasopharyngitis, otitis media, rhinosinusitis) in children and adolescents. Children with neutropenia and those with behavioral disorders had lower vitamin D levels and higher infection recurrence rates.

**What are the implications of the main finding?**

- Routine screening and correction of vitamin D status may reduce infection burden and support immune resilience in pediatric patients.
- Gender and immune status (e.g., neutropenia) should be considered when evaluating recurrent ENT infections and neurobehavioral vulnerability in children.

**Abstract:**

Background/Objective: Adequate serum vitamin D levels are essential for overall health, particularly in preventing oropharyngeal infections. This study aims to explore the relationship between serum vitamin D (25(OH)D3) levels and the prevalence of oropharyngeal diseases—specifically rhinosinusitis, otitis media, and nasopharyngitis—in patients at a private specialist medical clinic. Materials and Methods: The study involved 311 patients with an average age of 15.96 ± 15.06 years. Statistical analyses, including Student’s *t*-test and chi-square test, were conducted to evaluate the significance of the findings. Results: Nasopharyngitis was highly prevalent, affecting 83.27% of participants, with higher recurrence rates in those with lower vitamin D levels (*p* = 0.001). Otitis media was present in 53.37% of cases, while rhinosinusitis was less common and more frequently associated with higher vitamin D levels. Patients with behavioral disorders had significantly higher mean vitamin D levels (34.82 ng/mL ± 11.85) compared to those without (28.49 ng/mL ± 14.37) (*p* = 0.001). Conclusions: A subgroup of children with neutropenia (ANC < 1500/μL) exhibited significantly lower vitamin D levels and higher infection recurrence rates, underscoring their heightened vulnerability. This study highlights the importance of maintaining optimal vitamin D levels for pediatric health and suggests that higher vitamin D levels may reduce the risk of oropharyngeal infections.

## 1. Introduction

Vitamin D is a fat-soluble secosteroid essential for skeletal development, calcium-phosphate homeostasis, and immune regulation in children. Its deficiency has been associated not only with rickets and bone deformities but also with increased susceptibility to infections, particularly in the upper respiratory tract and oropharyngeal region [1,2,3]. Despite food fortification and supplementation programs, suboptimal vitamin D status remains prevalent in many populations, including otherwise healthy, non-critically ill children [4,5].

Vitamin D contributes to immune defense by enhancing the production of antimicrobial peptides such as cathelicidin and defensins, modulating inflammatory responses, and supporting mucosal barrier integrity [6]. Infections such as nasopharyngitis, otitis media, and rhinosinusitis are common in childhood and often recurrent, leading to frequent medical visits and antibiotic use. Several studies suggest that children with lower vitamin D levels experience a higher frequency of these infections [7,8].

Nutritional status, body composition, and lifestyle factors influence vitamin D levels. Higher body mass index (BMI) is inversely associated with serum vitamin D, possibly due to volumetric dilution and sequestration in adipose tissue [9,10,11]. Reduced outdoor activity, limited sun exposure, and dietary patterns low in vitamin D-rich foods contribute to this risk. Additionally, behavioral disorders in children may limit outdoor activities or influence diet quality, indirectly affecting vitamin D status.

Another group at increased risk of recurrent infections is children with neutropenia, defined as an absolute neutrophil count (ANC) < 1500/μL. While severe neutropenia is well studied in oncology patients, the implications of mild to moderate neutropenia in otherwise healthy children are less explored. Given vitamin D’s role in neutrophil function and innate immunity, deficiency in this subgroup may further amplify infection risk.

The COVID-19 pandemic has further highlighted the relevance of micronutrient sufficiency for immune resilience. Lockdowns and reduced outdoor exposure have been linked to declines in vitamin D levels globally, particularly in children, with potential downstream effects on infection susceptibility [12,13,14,15].

Existing literature supports the association between low vitamin D status and increased risk of respiratory tract infections [16,17,18], yet there is limited research specifically examining oropharyngeal infections in pediatric populations, especially when considering comorbid factors such as neutropenia and behavioral disorders. Furthermore, few studies have explored these associations in a clinical practice setting, where rapid testing and immediate supplementation decisions may influence outcomes.

Neutropenic children (absolute neutrophil count < 1500/µL) were included because even mild to moderate neutropenia, in the absence of oncologic disease, may impair innate immune defenses and predispose to recurrent respiratory tract infections. Given vitamin D’s role in modulating neutrophil function and enhancing antimicrobial peptide production, this subgroup provides a clinically relevant model for evaluating whether vitamin D deficiency amplifies infection vulnerability in pediatric patients [19].

Behavioral disorders such as inattention, anxiety, hyperactivity, and aggressiveness in children have been increasingly linked to systemic inflammation and neuroimmune dysregulation. Vitamin D plays a role in brain development, neurotransmitter synthesis, and modulation of inflammatory pathways, and deficiency has been associated with higher prevalence of mood and cognitive disorders in pediatric populations. Including behavioral disorders in our analysis allowed us to explore whether low vitamin D status may be a shared biological factor influencing both infection recurrence and neurobehavioral vulnerability.

Therefore, the present study aims to investigate the relationship between serum vitamin D (25(OH)D) levels and the prevalence and recurrence of nasopharyngitis, otitis media, and rhinosinusitis in children and adolescents attending a specialized ENT clinic. We additionally assess whether vitamin D status differs in subgroups with behavioral disorders or neutropenia, and whether demographic variables such as age, sex, BMI, and place of residence influence these associations. Our goal is to provide clinically relevant insights that can guide targeted prevention strategies, including screening and supplementation, to reduce infection burden and support overall pediatric health.

## 2. Materials and Methods

Between 2022 and 2023, a prospective study was conducted at a private otolaryngology (ENT) practice, in accordance with the guidelines outlined in the World Medical Association’s Declaration of Helsinki. The patient visited the private specialized medical clinic “OTORHINOMED” (Oradea, Romania) with their legal representative, reporting oto-rhino-pharyngeal issues (nasopharyngitis, otitis media, and rhinosinusitis). A clinical assessment was conducted. Each patient was followed by the ENT specialist for 6 months, with weekly visits during the acute phase or every 3 days in case of complications, and then monthly, from January 2023 to January 2024. Patients followed their general diet, except for those with severe malnutrition. Sun exposure was reduced or moderate for all patients.

Inclusion Criteria:

Patients aged between 0 and 18 years, presenting acute forms of oropharyngeal diseases, particularly nasopharyngitis, otitis media, and rhinosinusitis, were included in the study. Patients must have a confirmed diagnosis of acute oropharyngeal infections, specifically nasopharyngitis, otitis media, or rhinosinusitis. These diagnoses must adhere to the current clinical guidelines for acute infections, characterized by:

Nasopharyngitis: acute onset of nasal congestion, rhinorrhea, sore throat, and possible fever.

Otitis Media: sudden onset of ear pain, otorrhea, or hearing loss, with possible tympanic membrane inflammation observed during otoscopy.

Rhinosinusitis: acute inflammation of the nasal passages and sinuses, presenting with symptoms such as facial pain, nasal discharge, and congestion.

Exclusion Criteria:

Individuals aged over 73 years, those without vitamin D (25(OH)D3) measurements, individuals with diseases other than the specified acute oropharyngeal conditions, including chronic ailments that could impact serum vitamin D levels (e.g., osteoporosis), malnutrition, and those who declined participation were excluded from the study.

The study, involving 311 patients with an average age of 15.96 ± 15.06 years, aimed to examine the correlation between serum vitamin D levels and prevalent oropharyngeal diseases in children and adults, including rhinosinusitis, otitis media, and nasopharyngitis. Children with mild to moderate neutropenia (ANC < 1500/µL) were not excluded, provided they met the other eligibility criteria, because the study aimed to explore the role of vitamin D across different immune competence profiles. Including this subgroup allowed us to assess whether vitamin D deficiency could represent an additional modifiable risk factor for recurrent infections in already immunologically vulnerable children. The cohort was stratified into four groups based on the cumulative number of acute diseases as follows:

- A control group without any acute illness (0), with 32 persons (10.3%)
- A group with one acute disease (1), with 111 patients (35.7%)
- A group with two acute diseases (2), with 125 peoples (40.2%) and
- A group with all three cumulative acute diseases (3), with 43 persons (13.8%).

Each patient underwent testing using a rapid vitamin D test.

**Figure 1:**
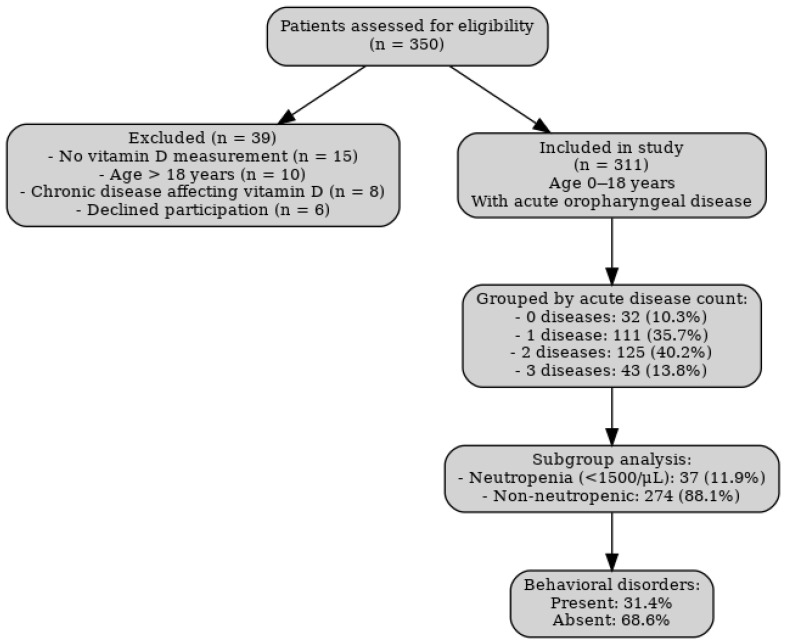
Flowchart of patient selection and eligibility criteria.

A detailed flowchart summarizing the patient selection process, applied eligibility criteria, and final study sample distribution is presented in Figure 1.

### 2.1. Clinical and Paraclinical Investigation

The clinical assessment took place at the medical office (OTORHINOMED, Romania), while the evaluation of paraclinical parameters was conducted at authorized laboratories. Paraclinical examinations included checking the blood pressure, blood sugar levels, and behavioral disorders.

Neutrophil counts were assessed through standard complete blood count (CBC) analysis for each patient. Children with neutrophil counts below 1500 cells/μL were identified as neutropenic and included in a subgroup analysis. These data were obtained at the initial evaluation, alongside vitamin D testing. Patients with neutropenia secondary to chemotherapy or chronic hematologic disorders were excluded.

Lack of concentration, anxiety, exhaustion, aggressiveness, and hyperactivity were considered behavioral disorders. The patients completed the anamnesis sheet, and if at least one of these disorders was present, they were included in the group with behavioral disorders. Furthermore, a thorough local clinical examination was performed, taking into consideration the comprehensive medical history. The clinical assessment utilized the Tanita MC780MA, a bioelectrical impedance body analyzer (BIA) sourced from Tokyo, Japan [20]. The obtained data were analyzed through the GMON 3.4.1 medical software developed in Chemnitz, Germany. Recognized by the World Public Health Nutrition Association (WPHNA), BIA body analyzers are renowned for their high accuracy and are frequently employed in assessing body composition. Up to the age of 18, BMI percentile was employed for assessment. The margin of error for the measurements was minimal, at 0.1 kg.

Behavioral disorders were assessed because of growing evidence connecting vitamin D deficiency with neuropsychiatric outcomes in children. By documenting these conditions alongside vitamin D status, we aimed to investigate possible correlations that could inform holistic management strategies, recognizing that immune and neurobehavioral health may be interconnected.

### 2.2. Rapid Vitamin D Test

For testing the vitamin D level, a rapid, cassette-type test can be utilized to semi-quantitatively detect 25-hydroxyvitamin D in whole blood using a fingerstick sample. The Vitamin D Rapid Test Kit employs a rapid chromatographic immunoassay to semi-quantitatively identify 25-hydroxyvitamin D (25(OH)D) in whole human blood. It provides a preliminary diagnostic test result and can be used to assess vitamin D deficiency. The JusChek (JusChek, Bucharest, Romania) rapid test was employed to conduct vitamin D testing and to colorimetrically determine the baseline vitamin D (25(OH)D) level. As a result, there are four categories for interpreting the results:

Insufficient: 0–20 µg/mL
Adequate: 21–29 µg/mL
Optimal: 30–55.5 µg/mL
Excessive: 55.5–150 µg/mL

The patients in the study initially did not receive regular vitamin D supplementation, possibly only for a short period, and their exposure to the sun was average or low, according to local statistics. After testing their vitamin D levels, supplementation was recommended where appropriate, based on age, with a reevaluation of vitamin D levels scheduled after 3 months.

### 2.3. Statistical Analysis

The data analysis utilized the Statistical Product and Service Solutions (version 20; IBM, Armonk, NY, USA) computer software program. Demographic variables, procedure frequency, and cost data obtained from the medical office were assessed for the two surveyed time points and across the two study groups to identify noteworthy trends. Calculations for means, frequency ranges, standard deviations, and tests of statistical significance were performed using Student’s *t*-test and the chi-square test. The Bravais–Pearson correlation coefficient gauged the relationship between the two variables. A level of *p* < 0.05 denoted statistical significance, while *p* < 0.01 indicated a high level of statistical significance. Post hoc analysis (Bonferroni) was implemented for additional subgroup analysis to scrutinize distinctions between groups.

## 3. Results

### 3.1. Demographic Description

The study, involving 311 patients with an average age of 15.96 ± 15.06 years, aimed to investigate the correlation between serum vitamin D levels and prevalent oropharyngeal diseases in children (rhinosinusitis, otitis media, and nasopharyngitis). The sample was assessed for skewness and kurtosis, with values falling between −3000 and 3000, and analyzed using a 95% confidence interval.

Table 1 presents the distribution of key clinical and biochemical parameters among pediatric participants, stratified by gender. The prevalence of nasopharyngitis, otitis media, and rhinosinusitis was high in both boys and girls, with no statistically significant gender differences observed. However, a greater proportion of girls had ≥2 cumulative diseases (21.6%) compared to boys (17.1%), approaching statistical significance (*p* = 0.065).

Notably, vitamin D deficiency or insufficiency (<30 µg/mL) was more common among females (24.8%) than males (23.2%), and this difference was statistically significant (*p* = 0.001). Interestingly, behavioral disorders were slightly more prevalent in boys (20.3%) than in girls (17.7%), with a significant association (*p* = 0.003), suggesting a possible gender-specific neuroimmune or psychosocial component.

These findings highlight the high burden of oropharyngeal disease and suboptimal vitamin D status in both sexes, with subtle but significant gender differences in behavioral vulnerability and vitamin D metabolism.

### 3.2. Description of Acute Diseases and Their Recurrence:

**Figure 2:**
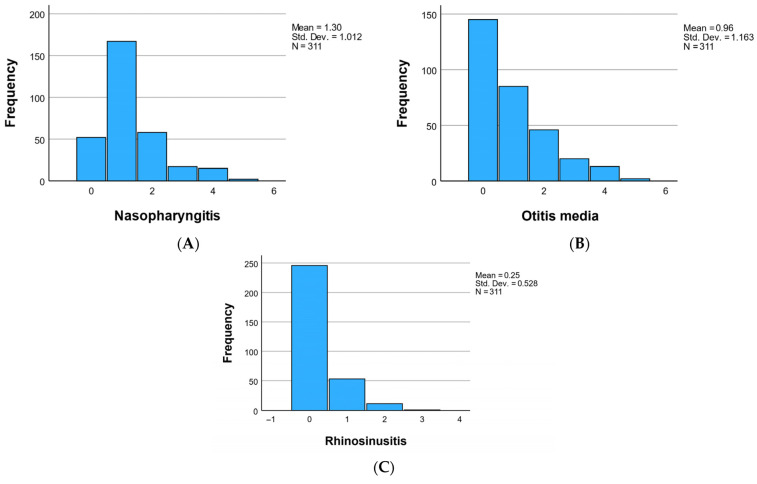
Frequency of nasopharyngitis (**A**), otitis media (**B**), and rhinosinusitis (**C**) at the cohort level in the last 6 months.

At the cohort level, the study meticulously monitored three acute diseases—namely, nasopharyngitis, otitis media, and rhinosinusitis—and tracked their recurrence over the preceding 6 months. Nasopharyngitis manifested in 259 individuals (83.27%) at least once, exhibiting a recurrence frequency of 1.30 ± 1.01, visually represented in Figure 2A. Otitis media, while less prevalent, was identified in 166 individuals (53.37%), displaying a recurrence frequency of 0.96 ± 1.16, as elucidated in Figure 2B. Rhinosinusitis occurred in 65 individuals (20.90%) at least once during the 6-month study period, with a recurrence frequency of 0.25 ± 0.53, visually depicted in Figure 2C.

### 3.3. Frequency of Diseases According to Gender

**Figure 3:**
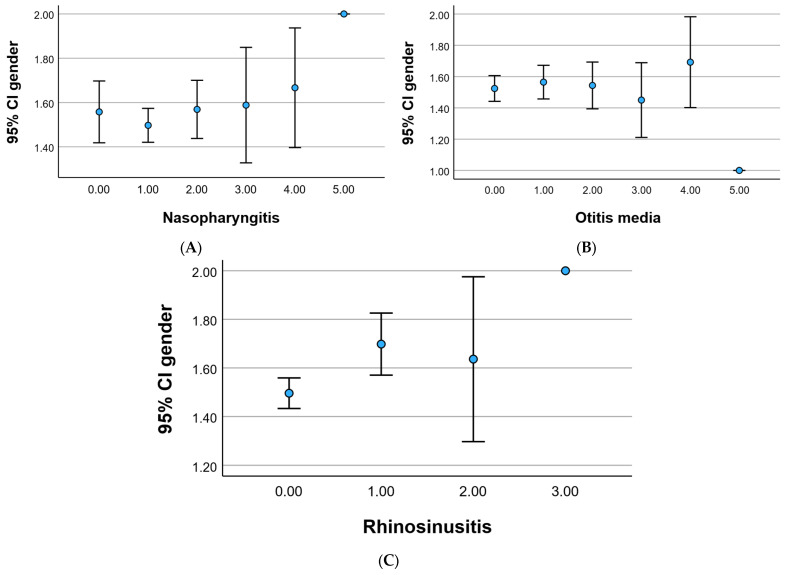
Recurrence frequency of diseases according to gender, including nasopharyngitis (**A**), otitis media (**B**), and rhinosinusitis (**C**).

The comprehensive depiction of disease distribution according to gender is visually presented in Figure 3. Intriguingly, the highest frequency of diseases is discerned among girls, with the exception of otitis, which exhibits greater prevalence in boys. An in-depth statistical analysis revealed that for the control group, differences between the sexes were statistically insignificant (*p* > 0.05). However, when delving into the recurrence frequencies of 3, 4, or 5, noteworthy and statistically significant differences (*p* < 0.05) emerged between males and females. This nuanced exploration underscores the gender-specific variations in disease occurrence and recurrence, providing valuable insights into potential predispositions or susceptibilities within the studied cohort.

For nasopharyngitis, 7.4% of males and 9.3% of females had no episodes, while 27.0% of males and 26.7% of females had one episode. A higher percentage of females experienced two episodes (10.6%) compared to males (8.0%). For otitis media, 22.2% of males and 24.4% of females had no episodes. A notable difference is that more females experienced one episode (15.4%) compared to males (11.9%). Regarding rhinosinusitis, 39.9% of males and 39.2% of females had no episodes. Females had a higher prevalence of one episode (11.9%) compared to males (5.1%).

### 3.4. Vitamin D Level by Cohort

Following serum vitamin D level testing, with an average value per cohort of 30.85 ± 13.81, the results were categorized into four groups based on the obtained values: 0–20 (deficiency), 20–29 (insufficient), 30–55.5 (optimal), and 55.5–150 (high). For a more accurate assessment of vitamin D levels by age category, the 0–18 years group was divided into two subgroups: 0–15 years and 16–18 years. Table 2 reveals a lower level of vitamin D in older individuals, with patients having the highest levels (average age of 6.80 ± 9.83), and these differences were statistically significant (*p* < 0.05). Statistically insignificant differences were observed between genders based on the vitamin D level category (*p* > 0.05), while a significant discrepancy was noted, with more individuals from rural areas facing vitamin D deficiency compared to those from urban areas (*p* < 0.05). It was observed that in the 16–18 years age category, as well as in the under 45 years category, no patient had an optimal level of vitamin D.

**Figure 4:**
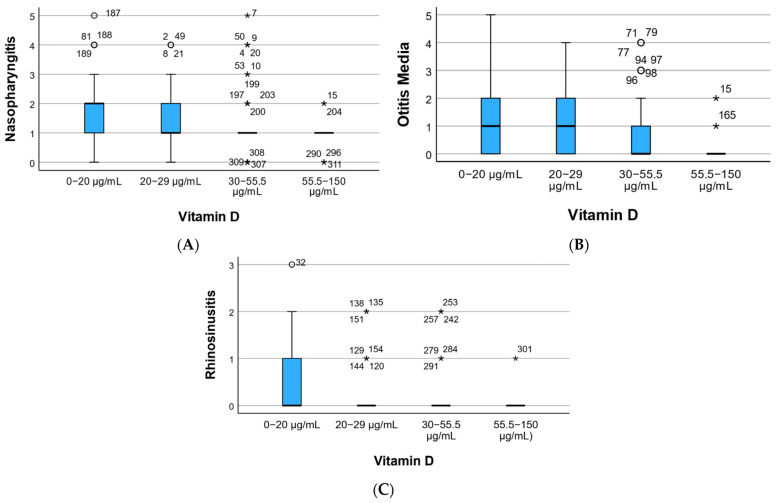
Visual depiction of rhinopharyngitis recurrence (**A**), otitis media recurrence (**B**), and rhinosinusitis recurrence (**C**) based on vitamin D levels. Circles (○) represent mild outliers (values 1.5–3 times the interquartile range [IQR] beyond the upper or lower quartiles). Asterisks (*) indicate extreme outliers (values > 3 × IQR from the quartiles). Numbers next to the symbols correspond to participant IDs.

Regarding disease recurrence based on vitamin D levels, Figure 4 illustrates a higher recurrence in nasopharyngitis for those with vitamin D deficiency (Figure 4A), recurrent otitis in individuals with low vitamin D levels (Figure 4B), and rhinosinusitis with recurrence 3 observed in the sole patient (Patient 1) with vitamin D deficiency (Figure 4C). The fewest recurrences were recorded at high vitamin D levels.

Following statistical analysis, the mean recurrence in nasopharyngitis was 1.91 ± 1.15 in individuals with vitamin D deficiency (0–20), significantly higher (*p* = 0.001) than 0.93 ± 0.59. The average frequency of otitis was highest at 1.38 ± 1.40 in those with deficiency and lowest at 0.20 ± 0.56 in individuals with high vitamin D levels (*p* = 0.023). Rhinosinusitis exhibited an exceptional case in those with a high vitamin D level, while more frequent recurrence was noted in individuals with vitamin D deficiency.

### 3.5. Neutropenia Subgroup

Among the pediatric participants, 37 children (11.9%) were identified as having neutropenia (ANC < 1500/μL). Within this group, the prevalence of nasopharyngitis was 94.6%, with a mean recurrence rate of 1.71 ± 0.89—significantly higher than non-neutropenic children (*p* = 0.002). Otitis media and rhinosinusitis were also more frequent, with 67.6% and 32.4% prevalence, respectively.

**Figure 5:**
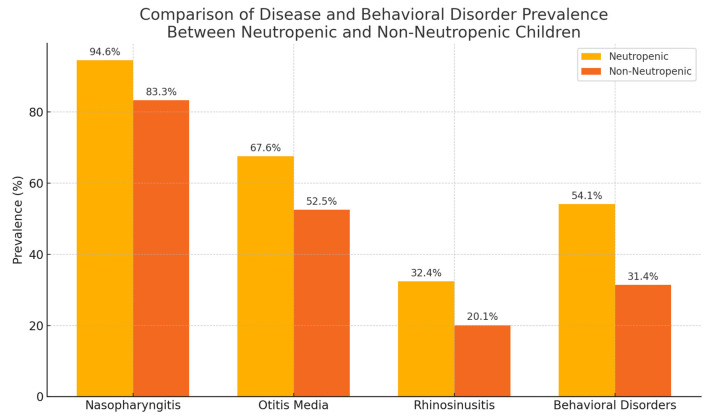
Prevalence of nasopharyngitis, otitis media, rhinosinusitis, and behavioral disorders in neutropenic versus non-neutropenic children.

Notably, the mean serum vitamin D level in neutropenic patients was 22.18 ± 9.75 ng/mL, significantly lower than in non-neutropenic peers (31.79 ± 13.51 ng/mL, *p* = 0.001). The majority (78.3%) of neutropenic children had vitamin D levels below 30 ng/mL. Behavioral disorders were present in 54.1% of neutropenic children compared to 31.4% of non-neutropenic children (*p* = 0.004) (Figure 5).

### 3.6. Cumulative Diseases

**Figure 6:**
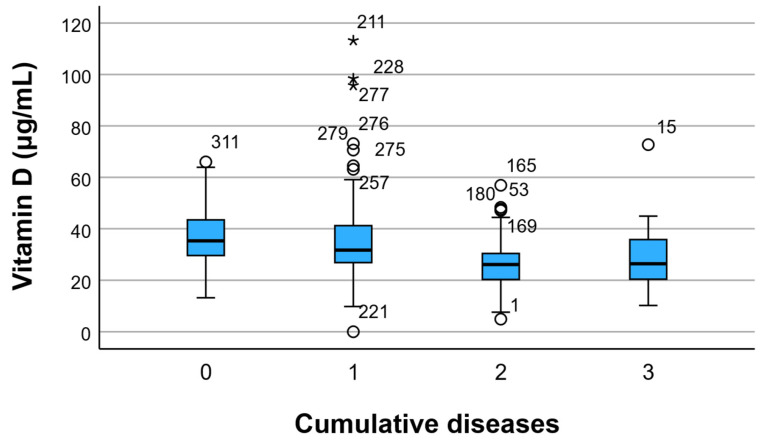
Visual representation of serum vitamin D levels based on the number of cumulative conditions. Circles (○) represent mild outliers (values 1.5–3 times the interquartile range [IQR] beyond the upper or lower quartiles). Asterisks (*) indicate extreme outliers (values > 3 × IQR from the quartiles). Numbers next to the symbols correspond to participant IDs.

Patients experiencing one, two, or all three of the acute conditions were monitored and assessed based on their serum vitamin D levels, as depicted in Figure 6. The highest value (36.66 ± 12.88) was observed in the control group comprising individuals without any of the tracked diseases. Simultaneously, a trend of decreasing vitamin D levels was noted: 35.54 ± 16.97 in those with only one of the tracked diseases, 26.08 ± 9.05 in individuals with two tracked diseases, and 28.29 ± 11.45 in those with all three conditions encountered in the last 6 months.

### 3.7. Description of Body Status

At the cohort level, the average BMI was recorded as 20.90 ± 7.04 kg/m^2^. In boys, the average BMI was 19.42 ± 6.44, while in girls, it was higher at 22.17 ± 7.31. It was observed that individuals from urban environments had a higher BMI (21.50 ± 7.34) compared to those from rural environments (20.21 ± 6.64).

**Figure 7:**
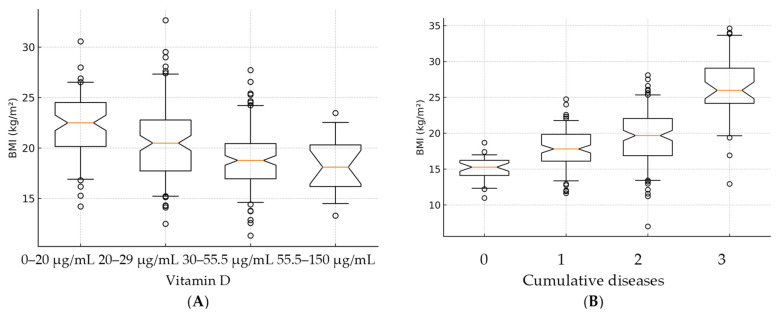
Relationship between BMI and vitamin D categories (**A**), as well as the association with the number of cumulative conditions (**B**). Open circles (○) represent outliers, defined as data points that fall outside 1.5 times the interquartile range (IQR) above the upper quartile or below the lower quartile. These indicate individual values that differ substantially from the central distribution.

When examining the average BMI based on the category of vitamin D (Figure 7A), a higher BMI was evident in individuals with insufficient and inadequate vitamin D levels, with no statistically significant difference between them (*p* > 0.05). Conversely, at adequate or optimal levels of vitamin D, a lower BMI was recorded, falling within the normal range.

In terms of the number of cumulative diseases, a statistically significant increase in BMI (*p* = 0.001) was observed in individuals with three cumulative diseases compared to those with no cumulative diseases (Figure 7B).

### 3.8. Behavior Disorders

The study analyzed the presence and absence of behavioral disorders according to age groups, the number of cumulative acute illnesses, and vitamin D levels.

Based on the number of cumulative illnesses, 5.8% of participants who did not have any illness had no behavioral disorder, while 4.5% had a disorder. In the single-disease group, 17.4% had no behavioral disorder, while 18.3% had a disorder. Among those with two diseases, 10.9% had no disorders, and 29.3% had behavioral disorders. Among those with three diseases, 3.2% had no disorders, and 10.6% had disorders.

**Figure 8:**
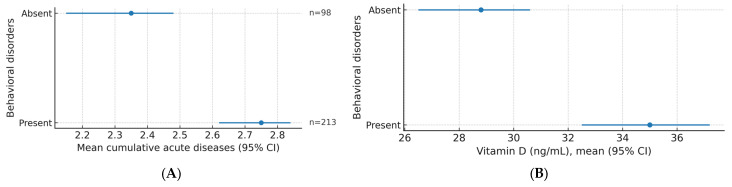
Relationship between behavioral disorders and (**A**) the mean cumulative number of acute diseases, and (**B**) serum vitamin D levels.

Regarding vitamin D levels, 3.5% of participants with a deficiency (0–20 ng/mL) had no behavioral disturbances, while 14.5% had disturbances. In the insufficient group (20–29 ng/mL), 10.9% had no impairments, and 23.8% had impairments. For the optimal level (30–55.5 ng/mL), 20.3% had no behavioral disturbances, and 22.2% had disturbances. In the high-level category (55.5–150 ng/mL), 2.6% had no disorders, and 2.3% had behavioral disorders (Figure 8).

### 3.9. Correlations

The Pearson correlation analysis revealed a statistically significant and inversely proportional relationship between serum vitamin D levels and nasopharyngitis, indicated by a negative Pearson coefficient (r = −0.263, *p* = 0.001). As serum vitamin D levels decrease, the recurrence frequency of nasopharyngitis significantly increases. A similar pattern was observed for the correlation between serum vitamin D levels and otitis media, with statistical significance and an inverse relationship, as indicated by the negative Pearson coefficient (r = −0.316, p = 0.001). In the case of rhinosinusitis, an inversely proportional relationship was recorded, although statistically insignificant (r = −0.109, p = 0.055).

**Figure 9:**
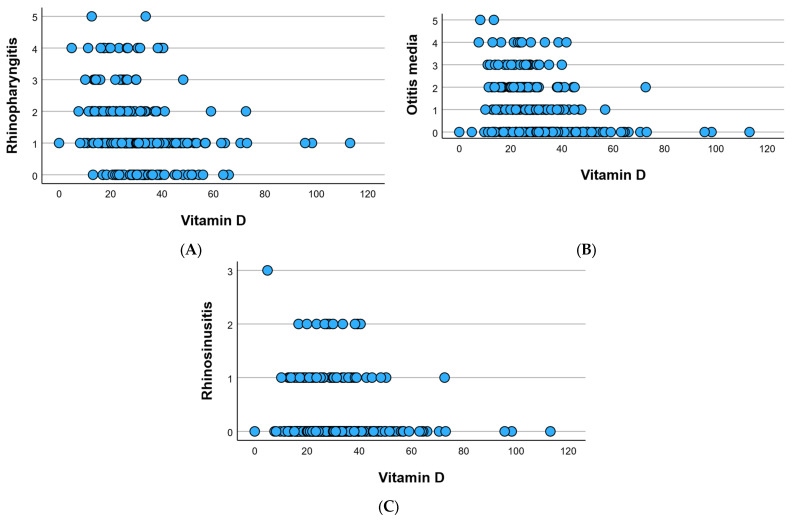
Visual representation of correlations regarding vitamin D and rhinopharyngitis (**A**), otitis media (**B**), and rhinosinusitis (**C**).

Regarding the correlation between cumulative diseases and serum vitamin D levels, a significant negative relationship was identified, suggesting that a decrease in vitamin D levels leads to the accumulation of diseases (r = −0.274, *p* = 0.001). These correlations are visually represented using the dot cloud technique in Figure 9.

Analysis using Pearson’s correlation revealed significant findings concerning the association between BMI and vitamin D, an inverse relationship was observed with statistical significance (r = −0.205, *p* = 0.001). This indicates a higher incidence of vitamin D deficiency in individuals with higher BMI. Consequently, it can be concluded that obesity has an impact on the serum level of vitamin D (Figure 9B).

**Figure 10:**
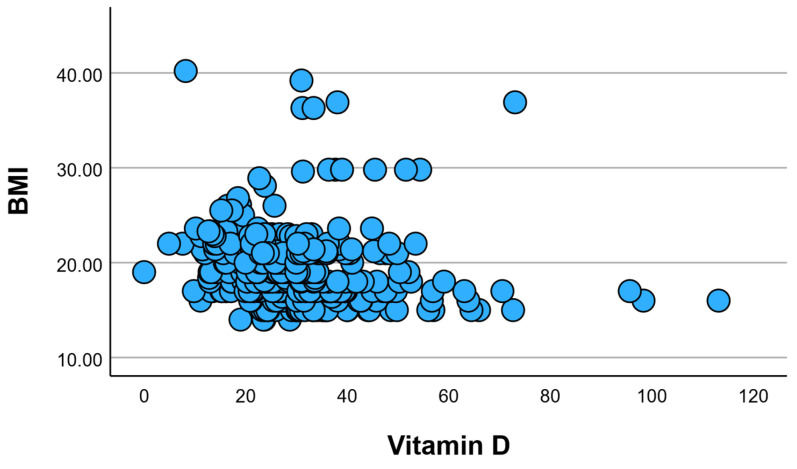
Visual representation of correlations regarding BMI and vitamin D.

In Figure 10 these correlations are visually depicted using the dot cloud technique, providing a clear representation of the observed relationships.

The study analyzed Pearson correlations between several factors and behavioral disorders (Table 3). The results are as follows: Vitamin D has a negative and significant correlation with behavioral disorders, with a correlation coefficient of −0.222 and a level of statistical significance (*p*) of 0.000. Age shows a positive and significant correlation with behavioral disorders, with a correlation coefficient of 0.152 and a *p*-value of 0.007. Body mass index (BMI) has a positive and significant correlation with behavioral disorders, with a coefficient of 0.174 and a *p*-value of 0.002. Nasopharyngitis did not show a significant correlation with behavioral disorders, with a coefficient of 0.110 and a *p*-value of 0.053. Otitis media has a positive and significant correlation with behavioral disorders, with a coefficient of 0.238 and a *p*-value of 0.000. Rhinosinusitis did not show a significant correlation, with a coefficient of 0.102 and a *p*-value of 0.072. Cumulative factors show a positive and significant correlation with behavioral disorders, with a coefficient of 0.240 and a *p*-value of 0.000.

## 4. Discussion

This study found a high prevalence of oropharyngeal infections in the pediatric population, with nasopharyngitis affecting 83.3% of participants, otitis media 53.4%, and rhinosinusitis 20.9% over a 6-month follow-up. Lower serum vitamin D levels were significantly associated with higher recurrence rates of nasopharyngitis and otitis media, and deficiency was more frequent among older children and those living in rural areas. Children with neutropenia had markedly lower vitamin D levels and a higher burden of recurrent infections compared with non-neutropenic peers. Behavioral disorders were also more common in individuals with lower vitamin D status and with multiple acute diseases. Additionally, an inverse correlation was observed between BMI and vitamin D, with higher BMI linked to lower vitamin D levels. These findings suggest that suboptimal vitamin D status may be a key modifiable factor in reducing infection recurrence and supporting immune resilience in pediatric patients, especially in vulnerable subgroups such as those with neutropenia or behavioral disorders.

Natural sources of vitamin D play a crucial role in childhood for the prevention of infections. Exposure to sunlight, consumption of vitamin D-rich foods like fatty fish, eggs, and fortified milk, and maintaining outdoor activities are key for sufficient vitamin D levels. Recent studies highlight the immunomodulatory effects of vitamin D, showing a significant reduction in the incidence of respiratory infections and other common childhood illnesses [16]. A 2023 study published in the *Journal of Pediatric Health* demonstrated that children with adequate vitamin D levels had a 30% lower risk of respiratory infections compared to those with deficiencies [17]. Another study from the *American Journal of Clinical Nutrition* in 2022 emphasized the importance of natural sunlight exposure, noting that young patients who spent more time outdoors had better overall immune function and fewer sick days [18]. Ensuring patients receive adequate natural vitamin D can significantly enhance their immune response and reduce infection rates, promoting better long-term health outcomes.

The study revealed a high prevalence of nasopharyngitis, affecting approximately 83.27% of individuals, with an average recurrence of 1.30 ± 1.01. This finding is consistent with previous research on nasopharyngeal carriage of Streptococcus pneumoniae, which indicated a high prevalence of nasopharyngeal colonization [21,22,23,24]. Additionally, the prevalence of nasopharyngeal carriage of respiratory pathogens was reported to be around 29% in another study, further supporting the high prevalence of nasopharyngitis.

In contrast, otitis media was found to have a lower incidence, affecting approximately 53.37% of individuals, with a mean recurrence of 0.96 ± 1.16. This lower incidence aligns with the lower prevalence of nasopharyngeal colonization by pneumococci in patients, which was reported to be 17% in a control group [25].

Rhinosinusitis was recorded at a lower percentage, diagnosed in 20.90% of the people studied, with an average recurrence of 0.25 ± 0.53. This finding is consistent with the meta-analysis of data from young children in China, which showed a high prevalence of nasopharyngeal carriage of Streptococcus pneumoniae, indicating a lower prevalence of rhinosinusitis compared to nasopharyngitis [24,26]. Consistent with the findings by García-Zendejas et al., who reported a strong association between Vitamin D deficiency and COVID-19 severity (OR = 6.13), our study similarly demonstrates that lower vitamin D status may contribute to increased susceptibility to recurrent oropharyngeal infections in children [27].

The subgroup analysis of children with neutropenia revealed a clear trend of more frequent oropharyngeal infections and significantly lower serum vitamin D levels. This is consistent with literature indicating that vitamin D enhances innate immune responses via upregulation of cathelicidin and defensins, both of which play a crucial role in neutrophil function. In neutropenic individuals, where phagocytic response is already compromised, the immunomodulatory benefits of vitamin D may be particularly critical. The higher burden of behavioral disorders in this group could also reflect the systemic effects of chronic low-grade inflammation and infection. Early recognition and correction of vitamin D deficiency in neutropenic children could thus provide dual benefits: reducing infection recurrence and improving neuroimmune balance.

The inclusion of neutropenic children was intentional, as this subgroup represents a clinically important population with compromised innate immunity, even outside oncologic or severe hematologic contexts. Neutrophils are essential for early pathogen clearance, and vitamin D has been shown to enhance neutrophil antimicrobial functions through the induction of peptides such as cathelicidin. By including children with mild to moderate neutropenia, we were able to evaluate whether vitamin D deficiency might further exacerbate infection risk in an already vulnerable immune profile. Our results, showing significantly lower vitamin D levels and higher infection recurrence rates in neutropenic participants, support the hypothesis that vitamin D insufficiency may act as a compounding factor in infection susceptibility in this group.

The study also indicated a trend towards a higher frequency of conditions in girls, with the exception of otitis, which was more common in boys. This observation aligns with the prevalence of nasopharyngeal carriage of S. pneumoniae by sex, which showed a slightly higher prevalence in males [24].

Rapid tests for vitamin D measurement, such as immunoassays and point-of-care devices, offer quick results but may lack the precision and specificity of standard methods like high-performance liquid chromatography (HPLC) or liquid chromatography-tandem mass spectrometry (LC-MS/MS). Standard methods are considered more accurate as they precisely detect various vitamin D metabolites, including the biologically active form 1.25-dihydroxyvitamin D (1.25(OH)2D).

The accuracy of rapid tests can be influenced by factors such as cross-reactivity and test calibration. This is particularly significant when measuring vitamin D3, as 1.25(OH)2D plays a crucial role in immune system modulation, influencing T cell activity and antimicrobial peptide production. Moreover, accurate assessment of vitamin D levels is vital due to emerging correlations between vitamin D deficiency and behavioral disorders. Studies suggest that low vitamin D levels may be associated with an increased risk of conditions such as depression, anxiety, and even cognitive impairments. Thus, precise measurement of vitamin D3 is essential for understanding its broader impacts on both physical and mental health.

Furthermore, the analysis of vitamin D levels suggested a significant correlation between vitamin D deficiency and the recurrence of nasopharyngitis and otitis media. This finding is consistent with previous literature reporting a higher prevalence of vitamin D deficiency among individuals with certain diseases, such as Graves’ disease and wheezing [28,29,30,31,32].

The observed direct correlation between BMI and age aligns with several studies in the specialized literature that have reported an increased prevalence of obesity among older individuals [33]. This finding supports the notion that age is a significant factor contributing to the incidence of obesity [34]. Additionally, our identification of an inverse relationship between BMI and vitamin D levels corroborates existing research highlighting the association between higher BMI and increased risk of vitamin D deficiency [35,36,37,38]. These consistent patterns underscore the relevance of considering age and BMI when assessing obesity trends and their potential impact on vitamin D status, providing valuable insights for public health interventions and personalized healthcare strategies.

In conclusion, the study’s findings on the prevalence of nasopharyngitis, otitis media, and rhinosinusitis, as well as the association between vitamin D levels and disease recurrence, are supported by existing research on nasopharyngeal carriage of pathogens and vitamin D deficiency.

Evidence linking vitamin D to infectious outcomes is mixed. For acute respiratory infections (ARIs), an earlier individual-participant meta-analysis suggested a small protective effect, particularly with daily dosing in deficient individuals; however, an updated 2025 meta-analysis of ~50 RCTs reported no statistically significant protection overall, and a 2024 review in older adults likewise found little to no difference in ARI incidence with supplementation [39,40].

Regarding tuberculosis, recent high-quality trials and syntheses have not shown preventive or consistent therapeutic benefits. Large prevention RCTs in Mongolian and South African schoolchildren found that long-term vitamin D supplementation did not reduce QuantiFERON conversion, TB disease, or ARI hospitalizations. Meta-analyses of adjunctive vitamin D during TB treatment show no overall effect on time to sputum culture conversion, with only genotype-specific signals in some studies. Collectively, these data indicate important null associations between vitamin D and TB risk or treatment response at the population level [41].

Implication for our findings: our pediatric ENT cohort addresses a different clinical phenotype (recurrent oropharyngeal infections) and care setting; thus, while our data suggest associations between lower vitamin D status and infection recurrence, we recognize that supplementation is not a universal anti-infective strategy, and benefits may depend on context (age, deficiency severity, dosing regimen, and comorbid immune vulnerability such as neutropenia). Future trials focused on clearly deficient pediatric subgroups are warranted [42,43].

Limitations of this study include its narrow focus on three acute diseases. Additionally, the absence of consideration for potential risk factors that may influence serum vitamin D levels represents another limitation. The study’s short duration can be regarded as a further constraint. An extended study, incorporating a group receiving vitamin D supplementation, would provide better control over the outcomes.

A notable strength of this study lies in emphasizing the significance of maintaining an optimal level of vitamin D in the context of acute diseases. This is underscored by the study’s findings, which suggest a potential role in reducing the frequency, recurrence, and cumulative burden of acute diseases.

Lack of concentration, anxiety, exhaustion, aggressiveness, and hyperactivity are considered behavioral disorders [44,45]. In the ENT specialty medical office, the availability of a rapid vitamin D test is very helpful. Although the accuracy is lower than that of laboratory biochemical analyses, this rapid test can be used to check for vitamin D deficiency using only capillary sampling. While the serum level of vitamin D is not typically monitored directly in ENT specialist offices, as are behavior disorders, it appears to be a useful tool for establishing correlations. These correlations can serve as guidelines for establishing procedures in the management of acute oropharyngeal infections.

The evaluation of behavioral disorders in relation to vitamin D was motivated by evidence that vitamin D receptors are widely expressed in brain tissue and influence neurodevelopmental and mood-regulating pathways. Deficiency has been linked to increased risk of attention-deficit/hyperactivity disorder, depression, and anxiety. In our cohort, lower vitamin D levels were significantly associated with higher prevalence of behavioral disorders, supporting the hypothesis that vitamin D may contribute to both immune resilience and neurobehavioral regulation in pediatric patients.

This pioneering research delves into the vitamin D status among people with non-critical illnesses, offering a comprehensive insight into potential advancements in pediatric healthcare. The objective is to develop universally applicable approaches for other healthcare systems. The review exposes current challenges in recognizing the interdependence and function of these systems in children’s and adult’s health, emphasizing the necessity for enhanced strategies. Further analysis may uncover additional themes, and thorough data collection would significantly contribute to a better understanding and improvement of children’s and adult’s healthcare.

## 5. Conclusions

The study shows a high prevalence of nasopharyngitis (83.27%) and otitis media (53.37%), with significant gender differences in recurrence rates, particularly higher in girls, except for otitis, which is more common in boys.

Vitamin D deficiency correlates significantly with recurrent nasopharyngitis and otitis media, and lower levels are found in older individuals, particularly in rural areas. Behavioral disorders are more common in younger individuals, those with more cumulative illnesses, and those with lower vitamin D levels.

The study also highlights that increasing age correlates with increased obesity, which is associated with lower vitamin D levels. Natural sources of vitamin D can help maintain optimal levels and reduce oropharyngeal infections.

In children with neutropenia, the combination of low neutrophil counts and vitamin D deficiency appears to amplify the risk of recurrent infections and behavioral disturbances. These findings suggest that targeted screening and correction of vitamin D levels in neutropenic patients may be a valuable adjunct in pediatric infection prevention strategies.

## Figures and Tables

**Table 1:** Key parameters in pediatric participants by gender (with *p*-values).

Parameter	Male (%)	Female (%)	*p*-Value
Nasopharyngitis ≥ 1 episode	65.8	66.8	0.242
Otitis Media ≥ 1 episode	54.2	56.3	0.132
Rhinosinusitis ≥ 1 episode	7.0	8.0	0.079
≥2 Cumulative Diseases	17.1	21.6	0.065
Vitamin D < 30 µg/mL	23.2	24.8	0.001 **
Behavioral Disorders	20.3	17.7	0.003 **

Statistically significant differences are marked with ** (*p* < 0.01).

**Table 2:** Vitamin D level by cohort.

Parameters		Vitamin D								F	*p*
		Deficient (0–20 µg/mL)		Insufficient (21–29 µg/mL)		Optimal (30–55.5 µg/mL)		Excessive (>55.5 µg/mL)			
		Count	%	Count	%	Count	%	Count	%		
Age	0–15 years	55	18.5	103	34.6	125	41.9	15	5.0	5.161	0.002 **
	16–18 years	6	20.7	8	27.6	15	51.7	0	0.0		
Gender	male	23	16.0	49	34.0	66	45.8	6	4.2	0.536	0.658
	female	33	19.8	59	35.3	66	39.5	9	5.4		
Environment	urban	42	25.1	55	32.9	64	38.3	6	3.6	4.510	0.004 **
	rural	14	9.7	53	36.8	68	47.2	9	6.2		

SD = standard deviation, F = coefficient ANOVA, *p* = statistically significance, ** = Correlation is significant at the 0.01 level (2-tailed).

**Table 3:** Pearson correlations between several factors and behavioral disorders.

Pearson Correlations		Vitamin D	Age	BMI	Nasopharyngitis	Otitis Media	Rhinosinusitis	Cumulative Factors
Behavior disorders	r	−0.222 **	0.152 **	0.174 **	0.110	0.238 **	0.102	0.240 **
	*p*	0.000	0.007	0.002	0.053	0.000	0.072	0.000
	N	311						

BMI = body mass index, r = Pearson, *p* = statistically significance, N = number of patients, ** = Correlation is significant at the 0.01 level (2-tailed).

## Data Availability

All the data processed in this article are part of the research for a doctoral thesis, being archived in the esthetic medical office, where the interventions were performed.
